# The Gut–Lung Microbiome Axis in Alveolar Stem Cell Regeneration and Lung Repair

**DOI:** 10.3390/microorganisms14071572

**Published:** 2026-07-18

**Authors:** Aotong Liu, Di Ran, Zekun Shen, Muhamed Rojba, Jilei Zhang

**Affiliations:** 1 Department of Pharmacology & Regenerative Medicine, College of Medicine, University of Illinois Chicago, Chicago, IL 60612, USA; aliu52@uic.edu (A.L.); zekun@uic.edu (Z.S.); 2 Division of Gastroenterology and Hepatology, Department of Medicine, College of Medicine, University of Illinois Chicago, Chicago, IL 60612, USA; diran@uic.edu; 3 Richard and Loan Hill Department of Biomedical Engineering, College of Engineering, University of Illinois Chicago, Chicago, IL 60612, USA; mrojba2@uic.edu

**Keywords:** gut–lung axis, gut microbiome, lung microbiome, dysbiosis, microbial crosstalk, microbial metabolites, lung injury, progenitor cell regeneration, alveolar epithelium

## Abstract

The mammalian respiratory system stands as a frontline barrier, constantly exposed to environmental insults, balancing defensive immunity with gas exchange. Historically considered sterile, the lung harbors a dynamic, low-biomass microbiome that evolves continuously in response to pulmonary pathologies. Accumulating evidence underscores that respiratory health and structural recovery are not autonomous but are critically integrated with distal microbial systems, especially the intestinal tract, through the gut–lung axis (GLA). This review characterizes the GLA as a bidirectional communication highway fueled by immune pathways, microbial metabolites, and direct microbial translocations. During acute or chronic injuries, such as COVID-19, COPD, asthma, idiopathic pulmonary fibrosis (IPF) and lung cancer, the gut microbiota serves as a remote metabolic “rheostat”. It delivers pivotal signaling molecules, such as short-chain fatty acids (SCFAs) and tryptophan metabolites (indoles), that could shape the local microenvironment in which the respiratory epithelium undergoes functional repair or maladaptive, fibrotic remodeling. Mechanistically, gut-derived butyrate enhances mitochondrial activity in alveolar epithelial cells, while resident progenitors, such as Alveolar Type 2 (AT2) cells, depend on intact mitochondrial fatty acid oxidation for proper regenerative differentiation. Conversely, critical lung illness disrupts this homeostasis via a “pathological circuit,” where severe pulmonary inflammation drives gut permeability, fecal dysbiosis, and the subsequent translocation of pathogen-associated molecular patterns (PAMPs, such as LPS) or gut-associated bacteria back into the pulmonary circulation. This review highlights the systemic nature of lung regeneration, which likely depends heavily on intestinal health through the GLA. Ultimately, leveraging these remote microbial networks through precision postbiotic supplementation, dietary priming, or microbiota transplantation represents a crucial frontier in precision medicine to promote definitive alveolar repair.

## 1. Introduction

The microbiome comprises all the microbes and their gene sequences, including homologous sequences, in a specific habitat at a specific time [1,2]. It includes all organisms, not just bacteria, but also archaea, fungi, and viruses. Various methods for obtaining DNA (metagenomics), RNA, metabolites, and proteins have been reported [3,4]. In the past, the oropharyngeal and gut microbiomes have been extensively studied, but the lungs have been considered sterile. However, this perception is changing thanks to advances in detection technologies, such as PCR, next-generation sequencing (NGS), and single-cell RNA/DNA sequencing [5,6]. Unlike the intestinal microbiome, the lung microbiome is mostly composed of bacteria, fungi, and viruses. But its composition and size change dynamically under the influence of different lung diseases, similar to the digestive tract [7,8]. For instance, pathogenic *Proteobacteria*, especially *Haemophilus*, were more prevalent in patients with asthma and COPD, whereas *Candida albicans* was more prevalent in patients with cystic fibrosis (CF) [9,10,11]. The lung microbiome has also been reported to influence lung development, as germ-free rodents show reduced lung parenchyma and less-developed alveoli [12]. Moreover, this lung microbiome is closely linked to the oropharyngeal and gut microbiomes because of their proximity, a relationship that has been extensively studied as GLA. The GLA is a bidirectional interaction between gut and lung microbes that influences the progression of intestinal and lung diseases through immune-related pathways (such as immune cells, cytokines, and chemokines), microbial products, metabolic pathways, and direct microbial transport.

The lungs are among the few internal organs exposed to a wide range of environmental pollutants, including organic, inorganic, and biological agents from diverse natural and anthropogenic sources. They are constantly at risk of developing simple to complex disorders, including acute and chronic lung injuries [13]. These injuries not only have distinct but also complex molecular mechanisms, processes, and tissue damage, but also undergo markedly different repair and regeneration. This lung regeneration is not just about the proliferation and differentiation of various cell types with progenitor capacity, but also involves contributions from neighboring cells and microenvironmental factors (e.g., the microbiome) through various molecular pathways [14]. Signaling pathways such as Wnt/β-catenin [15,16,17], Notch [18,19], and Yes-associated protein/Transcriptional co-activator with PDZ-binding motif (YAP/TAZ) [20,21,22] have been shown to contribute to lung regeneration. Meanwhile, microbiome-related pathways could also contribute to lung regeneration through the GLA. For instance, gut-derived SCFAs (e.g., butyrate) could enhance mitochondrial activity in alveolar epithelial cells and help preserve barrier integrity during injury [23], while AT2 progenitor cells themselves depend on intact mitochondrial fatty acid oxidation for proper regenerative differentiation, with disruption of this pathway driving aberrant, fibrosis-associated repair [24].

Several recent reviews have examined aspects of the GLA in the context of pulmonary disease [25,26,27,28]. These contributions have advanced our understanding of dysbiosis, immune crosstalk, and metabolic signaling along the GLA. However, existing reviews share a common limitation: they frame the gut–lung relationship primarily as a modulator of pulmonary inflammation, without addressing whether and how gut-derived signals determine the outcome of lung tissue repair. The fate of the injured lung is not determined at the peak of inflammation, but at the moment progenitor cells commit to either regenerative proliferation or maladaptive senescence. As emerging evidence suggests, this decision is governed epigenetically and metabolically by gut microbial signals. Furthermore, prior reviews have largely treated the three communication conduits of the GLA (immune cell trafficking, postbiotic metabolite signaling, and direct microbial translocation via the mesenteric lymphatic route) as parallel phenomena rather than as an integrated circuit whose balance determines lung progenitor cell fitness. For example, a recent review by Yang et al. [29] examined the lung–gut axis specifically in the context of pulmonary fibrosis, without extending this integrated-circuit framework to other major pulmonary diseases, explicitly linking it to alveolar progenitor cell regeneration, or examining the epigenetic and metabolic regulation underlying this process. Here, we address this gap by adopting the AT2 cell niche as an organizing framework to synthesize current evidence across five major lung diseases (COVID-19, COPD, asthma, IPF, and lung cancer) and to examine how GLA dysregulation compromises or supports the regenerative capacity of the lung epithelium. In this review, we aim to summarize our current knowledge of the role of the gut–lung microbial axis in lung damage caused by various diseases and in subsequent repair and regeneration.

## 2. Gut Microbiome

The microbiota colonizing our bodies is estimated to outnumber our body cells (approximately 38 trillion) [30], with the gastrointestinal (GI) tract harboring the majority of all human-associated microbes in the colon alone [3,31,32]. This microbial ecosystem develops through microbial acquisition during aging and functions as an “organ” that provides vital functions to the host, shaped by host factors, environmental cues, and microbial self-assembly rules [3]. Disruption of these functions, whether through dysbiosis or antibiotic treatment, can have significant consequences, including impaired immune development and colitis [33], underscoring the enormous taxonomic, genetic, and metabolic diversity of the gut microbiome and its interactions with the host [34]. Gut microbial dysbiosis, characterized by reduced and often unstable microbial diversity, is linked to numerous diseases affecting not only the intestine but systems beyond it, including the lungs via the GLA [35,36,37].

## 3. Lung Microbiome

The lung was long considered a sterile organ, a dogma debunked by the discovery of a resident lung microbiota using advanced detection technologies such as PCR, next-generation sequencing, and 16S rRNA sequencing [2,5,38]. Unlike the GI tract, the lung microbiome represents a much smaller, low-biomass community composed primarily of *Prevotella*, *Veillonella*, *Streptococcus*, and *Fusobacterium* species, reflecting the distinct environment of the alveolar space [7,28,39,40,41,42]. The upper and lower airways differ substantially in microbial composition: the upper airway (nasal passages, pharynx) is enriched with genera such as *Corynebacterium*, *Dolosigranulum*, *Moraxella*, and *Streptococcus*, whereas the lower airway is a comparatively unfavorable environment (limited nutrients, high oxygen stress) colonized primarily by anaerobic genera such as *Prevotella*, *Veillonella*, *Coprococcus*, and *Dorea* [38,43,44,45,46]. This lower-airway profile more closely resembles the oral microbiome than that of the gut, though it remains dynamically influenced by both and by lung health status [2]. This environment shifts during critical illness: altered alveolar oxygen concentration, pH, and nutrient availability drive lung dysbiosis [47,48]. In acute respiratory distress syndrome (ARDS), protein-rich alveolar fluid creates a gut-like, nutrient-favorable milieu, and the lung microbiome becomes enriched with gut-associated taxa such as *Bacteroidetes* and *Enterobacteriaceae* [28,49,50,51]. These findings support a causal role for the lung microbiome in pulmonary disease pathogenesis and underscore the bidirectional nature of the GLA, which influences intestinal and lung disease progression through metabolism, immunity, and microbial translocation.

## 4. Crosstalk Between the Gut and the Lung System

**Figure 1:**
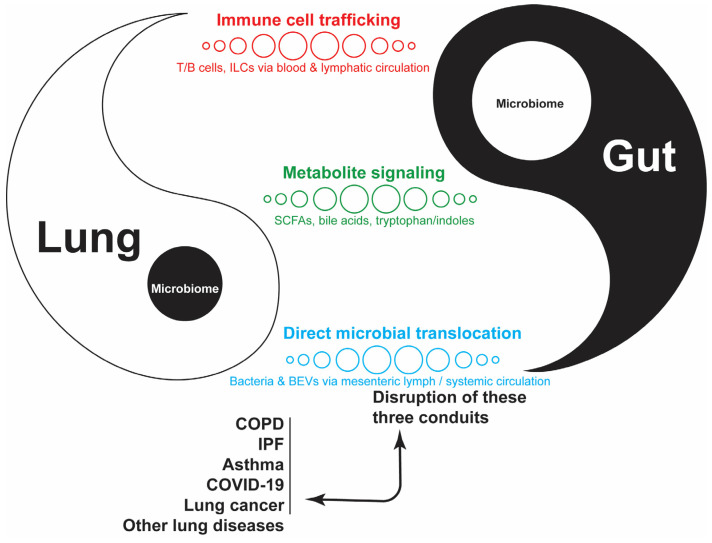
Overview of gut–lung microbial crosstalk. The gut and lung are shown as two interlocking, complementary compartments in a continuous bidirectional relationship. Following the classical Taijitu principle that each element contains the seed of its counterpart, a small “Microbiome” circle is nested within each compartment: the gut-derived microbiome within the lung compartment and the lung-derived microbiome within the gut compartment, which symbolizes that the two microbial communities are not independent but exist in mutual and bidirectional communication. Three mechanistic conduits connecting the two compartments are shown as distinct, color-coded rows, each corresponding to a mechanism: (i) immune cell trafficking (red), representing T cells, B cells, and innate lymphoid cells (ILCs) moving via the blood and lymphatic circulation; (ii) metabolite signaling (green), representing short-chain fatty acids (SCFAs), bile acids, and tryptophan/indole derivatives; and (iii) direct microbial translocation (cyan), representing bacteria and bacterial extracellular vesicles (BEVs) moving via the mesenteric lymphatic and systemic circulation. Within each row, the graduated circle sizes represent the associated pathway and bidirectional communications. The paired arrows connecting these conduits to the five disease contexts listed at the bottom (chronic obstructive pulmonary disease (COPD), idiopathic pulmonary fibrosis (IPF), asthma, COVID-19, lung cancer, and other lung diseases) indicate a bidirectional relationship: disruption of the three conduits can contribute to disease pathogenesis, and, reciprocally, disease can itself drive conduit disruption, a feed-forward relationship most directly documented in this review for ARDS/critical illness and COVID-19. The detailed, step-by-step immune and cytokine signaling cascade summarized at this systems level is shown in Figure 2.

It is widely acknowledged that lung and gut microbiomes are crucial for the local development and maintenance of immune homeostasis. Changes in their structure, quantity, and diversity of these microbiomes, known as dysbiosis, can compromise the immune barrier, trigger abnormal inflammatory responses, and increase the host’s susceptibility to various pathogens. Lung epithelium develops from ventral anterior foregut endoderm and is shaped by mesenchymal signals such as Fibroblast Growth Factor (FGF), Bone Morphogenetic Protein (BMP), Wnt, Retinoic Acid (RA), Hedgehog, and Notch, which are also key regulators of intestinal development [52]. This structural and biological foundation allows these two organs and their microorganisms to communicate through a sophisticated network of immune, endocrine, and neuroimmune pathways [28,39] (Figure 1, Table 1).

**Figure 2:**
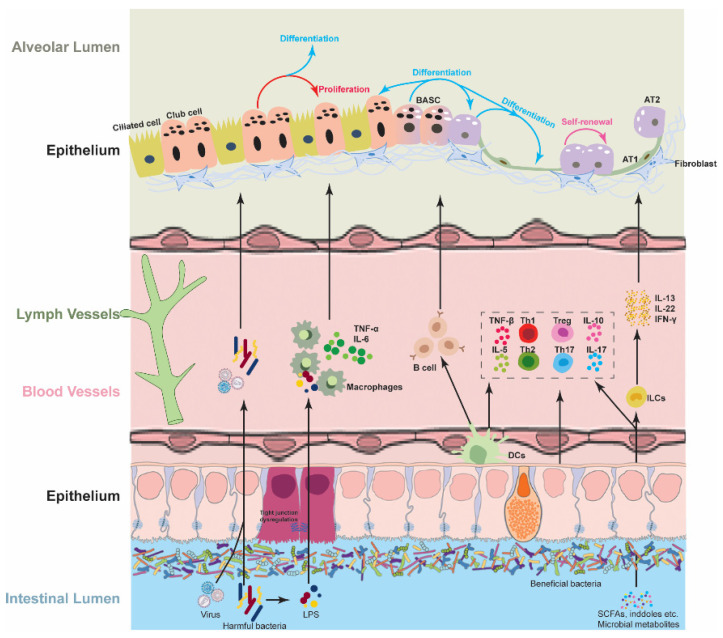
Diagram of possible mechanistic pathways to influence the regeneration of lung progenitor cells through the GLA, which is closely related to the blood system and lymphatic system. (1) Intestinal lumen: Harmful bacteria and viruses breach the gut epithelium at sites of tight-junction dysregulation (highlighted region), releasing lipopolysaccharide (LPS) and viral particles, while beneficial bacteria in the same lumen produce short-chain fatty acids (SCFAs), indoles, and other microbial metabolites. (2) Local immune activation: LPS activates macrophages to produce TNF-α and IL-6; dendritic cells (DCs) sample luminal antigens and interact with B cells; innate lymphoid cells (ILCs) stimulated by beneficial-bacteria-derived metabolites produce IL-13, IL-22, and IFN-γ. (3) Lymphocyte differentiation: DC and cytokine signaling drives differentiation of T-helper subsets (Th1, Th2, Th17) and regulatory T cells (Treg), with associated production of TNF-β, IL-5, IL-10, and IL-17. (4) Systemic transport: These microbial products, cytokines, and immune cell populations enter the blood and lymphatic circulation and are carried to the lung. (5) Alveolar epithelial response: Upon reaching the alveolar epithelium, this signaling supports proliferation of club cells and differentiation of bronchoalveolar stem cells (BASCs) toward alveolar type 2 (AT2) cells, which self-renew and further differentiate into alveolar type 1 (AT1) cells, alongside resident ciliated cells and adjacent fibroblasts. Collectively, the balance of pro-inflammatory (e.g., IL-5, IL-17) versus anti-inflammatory (e.g., IL-10) signals reaching the lung (set by the state of the gut microbiome and epithelial barrier) determines whether lung progenitor cells undergo productive regenerative differentiation or a response favoring ongoing inflammation and damage. This figure provides the mechanistic detail underlying the systems-level overview in Figure 1.

### 4.1. Immune-Related Interactions

#### 4.1.1. Immune Cells

The common mucosal immune system provides a primary mechanism for cellular crosstalk, where immune cells activated in the gut-associated lymphoid tissue (GALT) migrate to the bronchus-associated lymphoid tissue (BALT) via the systemic circulation and mesenteric lymphatics [80,81]. The T and B lymphocytes activated in the gut express specific homing receptors, such as α4β7 integrin and CCR9, allowing them to migrate directly to the lung. This imprinting is critically dependent on retinoic acid derived from vitamin A in gut dendritic cells, which drives α4β7/CCR9 expression and gut-homing phenotypes [82,83,84]. Once in the respiratory mucosa, B cells differentiate into plasma cells that produce secretory immunoglobulin A (sIgA), which has been implicated in the pathogenesis and progression of various lung diseases, thereby establishing a coordinated mucosal defense [85,86]. Moreover, gut-derived regulatory T cells (Tregs) and Th17 cells are critical for maintaining pulmonary homeostasis [28,87]. For example, butyrate, one of the short-chain fatty acids (SCFAs), can promote Foxp3+ Treg differentiation to alleviate allergic airway inflammation [88].

These gut microbiomes not only influence adaptive immune cells but also “prime” lung-resident innate cells. It has been reported that intestinal-derived metabolites, such as propionate, can reprogram alveolar macrophages (AMs), altering their inflammatory responses and phagocytic capacity during lung injury [73]. Furthermore, recent studies have identified a novel subtype of gut-derived type II innate lymphoid cells (inflammatory ILC2s, iILC2s) that migrate to the lung and play a pivotal role in the pathophysiology of asthma [89,90]. There is growing evidence that antigen and microbial cues in the GALT influence the migration of T cells, ILCs, and B cells to the lung, which can either cause damaging inflammation or promote resolution and healing, depending on microbial health and the Treg/Th17 balance. Microbial imbalance in the gut can weaken barrier integrity, change SCFA production, and shift the Treg/Th17 balance toward a Th17-dominant, hyperinflammatory lung response, thereby worsening ALI/ARDS and sepsis-related respiratory failure [28,74,91,92]. These changes were also seen in neonatal primate models with antibiotic-induced dysbiosis and could be partially corrected with fecal microbial transplantation (FMT) [93]. These FMT benefits can also be achieved through microbiota-supportive strategies, such as dietary fiber/SCFAs and probiotics, which can enhance Tregs and improve lung outcomes in asthma, sepsis, and ARDS failure [28,74,91,92].

#### 4.1.2. Immune Cytokines and Chemokines

In our immune system, cytokines and chemokines act as molecular messengers, converting microbial signals into widespread inflammatory or anti-inflammatory responses in the GLA. Intestinal dysbiosis or “leaky gut”, characterized by increased gut permeability, facilitates the translocation of lipopolysaccharides (LPSs) and other pathogen-associated molecular patterns (PAMPs). These trigger TLR4-mediated signaling cascades, most notably the NF-kB pathway, leading to the production of proinflammatory cytokines such as IL-6, TNF-α, and IL-1β in the lung [73,74,75]. When entering the bloodstream and reaching the lungs, lipopolysaccharides (LPSs), a component of Gram-negative bacterial walls, activate the TLR4/MyD88/NF-kB pathway and initiate a pro-inflammatory response that characterizes ARDS and severe COVID-19 [76,77,78]. In severe asthma, gut-derived LPSs have been shown to induce elevated oncostatin M (OSM) expression through the TLR4-MyD88 pathway, which drives mucus hypersecretion [27,79]. More broadly, gut-microbiota-targeted interventions have been shown to modulate systemic inflammatory disease via TNF-α suppression and microbiota regulation in other organ systems as well [94], illustrating that this immune-modulatory principle extends beyond the lung. Conversely, beneficial metabolites (such as SCFAs) bind to G-protein-coupled receptors (GPCRs), including GPR41, GPR43, and GPR109A, on lung cells [53,54]. This interaction promotes the release of cytokines such as IL-10 and IL-18 and enhances IL-22 production by type 3 innate lymphoid cells (ILC3s), which is crucial for maintaining the integrity of the respiratory epithelial barrier [95,96,97].

As we mentioned earlier, the GLA is a bidirectional communication where intestinal hyperpermeability both results from and exacerbates lung inflammation, as discussed in reviews of sepsis, ARDS, COPD, COVID-19, and pulmonary fibrosis [26,28,92,98,99,100]. In ARDS and ventilator-induced lung injury, pulmonary cytokines (TNF-α, IL-1β, and IL-6) could reach the intestine via the circulation, which has been tightly linked to disruption of intestinal tight junctions (e.g., claudins, occludin, ZO-1), MLCK activation, and increased intestinal permeability [28,101,102].

### 4.2. Microbial Products

The metabolic output of the gut microbiota functions as a systemic signaling mechanism, connecting the intestinal lumen to the alveolar space. These small molecules, or “postbiotics,” enter the portal and systemic circulation, directly influencing the lung’s inflammatory state and its ability to repair epithelium. Among these, SCFAs, bile acid derivatives, tryptophan metabolites, and bacterial extracellular vesicles have received the most attention because they link intestinal microbial activity to immune regulation and tissue repair.

#### 4.2.1. Short-Chain Fatty Acids (SCFAs)

Short-chain fatty acids (SCFAs), mainly acetate (C2), propionate (C3), and butyrate (C4), are primarily (90%) absorbed in the intestine and are present in the molar ratio of 60:25:15 in the colonic lumen. Smaller amounts of other SCFAs, such as formate, valerate, and caproate, are also produced. However, this SCFA ratio can vary depending on the composition of the intestinal microbiota, diet, fermentation site, and host genotype. The fermentation of dietary fibers mainly occurs in the proximal colon, which hosts a highly diverse and stable bacterial community with the greatest substrate availability (e.g., *Bacteroides* and *Faecalibacterium prausnitzii*) [54,103].

As noted earlier, SCFAs can bind to G-protein-coupled receptors (GPCRs), specifically GPR41 (FFAR3), GPR43 (FFAR2), and GPR109A (HCAR1), which are present on lung immune cells and alveolar epithelial cells [53,54,55]. Activating these receptors has been shown to inhibit LPS-induced inflammatory signaling and decrease TNF-α and edema in models of acute lung injury [55,102,104]. Additionally, supraphysiologic SCFA levels (10–25 mM) were reported to enhance TNF-α-induced IL-6 and CXCL8 in human lung fibroblasts and airway smooth muscle (ASM) via FFAR3-p38 MAPK signaling [56]. These findings indicate that SCFA effects on lung tissue are concentration- and context-dependent: physiologic concentrations acting through GPR41/GPR43/GPR109A appear predominantly anti-inflammatory and pro-regenerative, whereas supraphysiologic concentrations can paradoxically potentiate inflammatory signaling in structural lung cells. As a histone deacetylase (HDAC) inhibitor, butyrate has been found to increase histone acetylation and promote the differentiation of regulatory T (Treg) cells in the intestine [105,106]. Whether this same HDAC-inhibitory mechanism extends to alveolar epithelial cells and shapes a pro-regenerative AT2 gene expression program directly has not yet been established, and represents a plausible but untested extension of this pathway to the lung. Separately, metabolically active gut microbiota that produce SCFAs can transmit LPSs and SCFAs to the lungs, thereby creating a primed lung immunometabolic tone [73]. Similarly, hematopoiesis of dendritic cell precursors in the bone marrow can be influenced by elevated circulating propionate levels, favoring a non-allergic immune phenotype in the lungs [57]. It is worth noting that AT2 regeneration requires intact fatty acid oxidation and acetyl-CoA, which can be restored with acetate supplementation, thereby further normalizing abnormal repair processes in AT2 cells [24]. This supports the idea that SCFAs can act as metabolic substrates shaping epigenetic and regenerative programs in lung progenitors.

SCFAs, mainly acetate, propionate, and butyrate, are among the best-characterized gut-derived metabolites with potential relevance to lung repair. Overall, the available data support SCFAs as metabolic and epigenetic regulators that may help determine whether injured lung epithelium undergoes effective regeneration or maladaptive remodeling, with their net effect shaped by local concentration and cellular context rather than being uniformly protective in all settings.

#### 4.2.2. Bile Acids and Tryptophan Metabolites

Primary bile acids (e.g., cholic acid (CA), chenodeoxycholic acid (CDCA)) are produced from cholesterol in the liver, then conjugated and released into the gut. A small amount reaches the colon, where bacteria, including *Clostridium scindens* and related *Firmicutes*, deconjugate and 7α-dehydroxylate them via bai operons, forming secondary bile acids (e.g., primarily deoxycholic acid (DCA), lithocholic acid (LCA)) [58]. These can interact with the FXR (Farnesoid X receptor) and TGR5 (Takeda G-protein-coupled receptor 5), which have been shown to reduce inflammation, vascular remodeling, and fibrosis in experimental models of pulmonary hypertension and fibrosis using bile acid-derived agonists [59].

In contrast to this systemic, largely protective FXR/TGR5 signaling axis, direct local exposure of the lung to high concentrations of bile acids represents a distinct, potentially injurious mechanism. Exposure to high localized concentrations of lithocholic acid (LCA), deoxycholic acid (DCA), and chenodeoxycholic acid (CDCA) in the lung (e.g., microaspiration) can be harmful, such as causing alveolar epithelial injury, epithelial–mesenchymal transition, and fibroblast activation via FXR-dependent and independent pathways in vitro and ex vivo IPF tissue [60].

On the other hand, gut bacteria such as *Lactobacillus*, *Bifidobacterium*, *Peptostreptococcus*, and *Clostridium* spp. metabolize dietary tryptophan into indole derivatives, many of which are Aryl Hydrocarbon Receptor (AhR) ligands [61,62]. These microbial Trp catabolite-induced AhR signals support barrier integrity, IL-22 production, and antimicrobial defense in the intestine, and these same pathways are involved in respiratory protection [61,62,63]. Indole-3-aldehyde from *Lactobacillus* activates the AhR/IL-22 axis, promoting epithelial protection and antifungal resistance at mucosal surfaces [64,65]. It is also being studied for lung diseases like cystic fibrosis [66,67]. Indole-3-aldehyde also directly reduces lung inflammation in COPD models by activating pulmonary AhR and inhibiting HDAC5/6 (histone deacetylase)-NF-κB-NLRP3 (Nod-like receptor pyrin domain 3) signaling, thereby lowering cytokine levels and tissue damage [68]. In a gut–lung model, dietary tryptophan or indole, along with *Lactobacillus plantarum*, reversed antibiotic-induced defects in lung defense, restored alveolar macrophage phagocytosis, and reduced the *Pseudomonas aeruginosa* burden through intestinal AhR–IKKβ-ROS signaling [63].

#### 4.2.3. Bacterial Extracellular Vesicles

Bacterial extracellular vesicles (BEVs) are nanoscale vesicles released by both Gram-positive and Gram-negative bacteria that contain proteins, lipids, DNA/RNA, and pathogen-associated molecular patterns (PAMPs). These BEVs cross the intestinal epithelium via transcytosis or paracellular pathways and then enter the portal and systemic circulations, reaching tissues outside the intestine. They have been identified as a common mechanism for microbiota–organ communication and systemic disease regulation [107,108,109].

Gut-derived commensal/probiotic (e.g., Bifidobacterium) BEVs could alter cytokine and oncogenic pathways in lung tumors by increasing CD8 T-cell infiltration and enhancing the efficacy of anti-PD-1, suggesting remote gut-lung communication via BEVs [70]. Researchers also highlighted that commensal BEVs may help maintain a tolerogenic, infection-resistant lung immune landscape [71,72]. Whether circulating gut microbial metabolites physiologically protect the lung, support surfactant homeostasis, or drive alveolar fluid clearance has not yet been directly demonstrated. Because comparable pro-regenerative and edema-clearing effects of these same metabolite classes have been reported in other tissues via modulation of immune and inflammatory pathways, we propose this as a testable hypothesis and a priority direction for future GLA research, rather than an established mechanism.

### 4.3. Direct Microbial Transportation

As the richest and most complex microbial community in the human body, the gut microbiota comprises trillions of microorganisms, including bacteria, archaea, fungi, viruses, and protozoa [39]. Although the respiratory tract has a less abundant and diverse microbiota than the GI tract, it hosts multi-species microbial communities. These microbes can crosstalk directly by transporting between the gut and lungs via systemic dissemination, the mesenteric lymphatic system, and the oral–gut–lung continuum, especially under dysbiosis conditions with a disrupted epithelial barrier and mucosal immune system.

Oralome, or the oral microbiome, comprises over 700 microbial species in the oral cavity, making it the second-largest microbial community in humans after the gastrointestinal tract [45]. It is not surprising that these microbiomes play a vital role in maintaining pulmonary immune homeostasis via the oral–lung axis, considering the anatomical connection between the oral cavity and the respiratory tract. For instance, the oral cavity is an active site of infection and serves as a reservoir for pathogens involved in respiratory diseases [110]. Additionally, a small amount of SCFAs derived from the oralome has been shown to affect the host immune response in various lung diseases [111,112]. Furthermore, the oral cavity and upper airway function as a metaphorical “microbial gatekeeper” to safeguard respiratory health [113]. The oral, upper airway, and gut microbiota each establish and maintain a highly interactive microbial community with the lower airway microbiome, and highly dynamic microbial interactions likely occur in the oral cavity, airways, and gut. These bidirectional communications through microbial translocations have been highlighted in diseases such as colorectal cancer and COVID-19 [114,115]. In acute lung injury (ALI) and acute respiratory distress syndrome (ARDS), cytokines produced from pulmonary inflammation can circulate and disrupt intestinal epithelial integrity, leading to intestinal hyperpermeability. This causes gut microbes, such as *Bacteroidetes* and *Enterobacteriaceae*, to translocate across the intestinal mucosa and even enter the lung [50,51]. Conversely, in DSS-induced colitis models, the loss of intestinal tight-junction proteins and increased FITC-dextran permeability coincide with the translocation of GFP-labeled gut bacteria into the lungs, where they induce IL-17-driven neutrophilic inflammation and airway hyperresponsiveness [75].

The intestinal microbiome can be directly transferred to the lung via the mesenteric lymphatic network, one of the two parallel systems for intestinal outflow drainage, along with the portal venous circulation. Interstitial fluid from the gut enters lacteals, travels through the collecting mesenteric lymphatics, the cisterna chyli, and the thoracic duct, then empties into the left subclavian vein. This process allows gut-derived lymph to reach the right heart and pulmonary circulation before any hepatic filtration. This lymph flow bypasses Kupffer cells and first-pass metabolism, enabling harmful molecules or microbiota to reach the lung even when portal blood appears relatively free of bacteria and endotoxin [116,117,118]. Once delivered to the pulmonary microcirculation, mesenteric lymph-borne mediators prime and recruit neutrophils, increase endothelial permeability, and activate resident macrophages and epithelial cells [119,120,121,122,123,124]. In the colon ascendens stent peritonitis (CASP) models, septic mesenteric lymph was enriched with TNFα, IL-1β, IL-6, and phospholipase A2, which promote endothelial apoptosis and barrier disruption. Draining this lymph reduces cytokine expression, preserves pulmonary microvascular endothelium, and decreases edema [120]. Across sepsis and trauma models, the resulting ALI exhibits protein-rich edema, polymorphonuclear sequestration, and diffuse alveolar damage, creating an environment that is likely to hinder AT2s-mediated epithelial regeneration, even though direct AT2-specific data remain limited [116,117,118].

## 5. Contributions of the Microbial Gut–Lung Axis to Lung Damage and Regeneration

The primary role of the mammalian respiratory system is to exchange gases with the blood, which is then circulated throughout the body by the cardiovascular system. As the frontline barrier to inhaled particles, pathogens, and environmental toxic chemicals, the lung epithelium forms a multicellular, immune-primed epithelial barrier and is populated by a powerful immune system composed of hematopoietically derived myeloid and lymphoid cells [125]. This setup serves as a “first contact” for sensing and defending against microbial invasion and environmental pollutants. The respiratory system is divided into two primary functional parts: gas-conducting tubular airways and gas-exchanging alveolar sacs, with cell composition and function varying significantly along the proximal-to-distal axis of the respiratory tract [125,126,127]. The proximal airways start in the trachea and bronchi, then branch into smaller airways called bronchioles. The airway lining includes basal cells, which act as stem cells; goblet cells, which produce mucus; and ciliated cells, which move mucus and particles. Further along, secretory club cells are found [128,129]. The distal lung contains millions of alveoli that form a honeycomb-like structure, providing a vast surface area for gas exchange. Alveoli are lined with thin, flat, squamous alveolar type 1 (AT1) cells, which account for over 95% of the gas-exchange surface and are responsible for gas exchange [128,130]. They also contain cuboidal alveolar type 2 (AT2) cells, which make up only 2–5% of the alveolar surface but are nearly twice as numerous as AT1 cells. These AT2 cells perform multiple roles by secreting surfactant, maintaining fluid balance, and regulating immune responses, while also functioning as stem cells involved in lung homeostasis, repair, and regeneration [128,131].

Studies have shown that the mechanism of tissue repair varies considerably depending on the extent of damage (acute vs. chronic), the site of damage (airway vs. alveoli), and the type of damage-causing agent (pathogen, particle, or toxin) [126]. The importance of these regenerative capabilities has been underscored by the COVID-19 pandemic, which caused acute lung injury in millions worldwide, and by chronic obstructive pulmonary disease (COPD), the third leading cause of death globally [125,132]. These strategies for repairing and regenerating structures and lost cells are not limited to local lung tissues but extend beyond, including the GI tract through the GLA (Figure 2, Table 1 and Table 2).

The lung, despite remaining generally quiet, has a remarkable ability to regenerate, with resident progenitor cell populations dividing and differentiating into various cell types in response to injury [158]. Lung regeneration is a systemic process where the gut microbiota functions as a distant metabolic “rheostat.” Beyond merely acting as a barrier, microbial signals determine whether the lung undergoes functional repair or maladaptive remodeling. As we mentioned earlier, gut-derived SCFAs support mitochondrial activity in alveolar epithelial cells, while AT2 progenitor cells rely on intact mitochondrial fatty acid oxidation for proper regenerative differentiation [23,24]. Furthermore, microbial tryptophan metabolites (e.g., indoles) serve as ligands for the AhR on the alveolar surface. AhR activation in alveolar epithelial cells has been shown to preserve epithelial identity and limit TGF-β-driven epithelial-to-mesenchymal transition during injury, while enhancing barrier function and suppressing inflammatory cytokine expression [69]. Complementing this epigenetic mechanism, a recent high-throughput organoid screen identified AHR activation in the pulmonary mesenchyme, rather than the epithelium directly, as sufficient to resolve TGF-β1-induced fibroblast activation and reduce accumulation of Fn1+ Krt8+ ADI cells, thereby promoting productive AT2-to-AT1 differentiation over the pathological transitional state [159]. This finding adds a stromal, cell-non-autonomous dimension to the AT2 fate-switch mechanisms discussed above, and raises the question of whether gut-derived AhR ligands act similarly on the pulmonary mesenchyme, not just the epithelium, to shape AT2 regenerative outcomes.

Across disease states, the central question is not simply whether the microbiome is altered, but whether that alteration changes the regenerative trajectory of the injured lung. Therefore, each disease section below should be interpreted as a disease-specific test case for the broader concept that gut-lung microbial signaling influences progenitor-cell fitness, epithelial repair, and fibrotic remodeling.

### 5.1. COVID-19

Caused by the highly pathogenic severe acute respiratory syndrome coronavirus 2 (SARS-CoV-2), coronavirus disease 19 (COVID-19) has infected more than 770 million people and resulted in over 6.9 million deaths worldwide as of 2024, since the first case was reported in 2019 [160]. This virus primarily targets respiratory tract cells, such as nasal epithelial cells and AT2 cells, because they express viral entry factors, including TMPRSS2 (Transmembrane protease, serine 2) and ACE2 (angiotensin-converting enzyme 2), which are regulated by the gut microbiota in the colon [115,161,162]. Under severe circumstances, it can cause pneumonia and acute respiratory distress syndrome (ARDS), which is characterized by noncardiogenic pulmonary edema, bilateral pulmonary infiltrates, and profound hypoxemia leading to respiratory failure [162]. The acute phase of ARDS results from endothelial injury and diffuse alveolar damage, while the late phase is characterized by impaired proliferation of AT2 cells and fibroblasts, followed by chronic inflammation and extensive alveolar fibrosis, leading to loss of normal lung architecture [163]. These alterations have been linked to the microbiomes of the lung and the digestive system. For instance, COVID-19 patients with an increased pulmonary microbial burden have a lower probability of recovery from invasive mechanical ventilation and a higher mortality rate [133,134]. The lung microbiome composition is associated with changes in TNF-α, and microbial factors may activate inflammasomes, leading to IL-1β release, which has been widely reported to play a critical role in AT2 proliferation and differentiation [135,136,137]. Similarly, intestinal dysbiosis in patients infected with SARS-CoV-2 has been associated with progression and severity of COVID-19 and is characterized by reduced numbers of anti-inflammatory bacteria, such as *Bifidobacterium* and *Faecalibacterium*, and reduced numbers of butyrate producers, including several genera from the *Ruminococcaceae* and *Lachnospiraceae* families [39].

Although SARS-CoV-2 has been shown to infect both AT1s and AT2s ex vivo, it primarily targets AT2s and ciliated airway cells [164,165,166]. However, reports have indicated the enrichment and significance of various progenitor cell types in regenerating damaged respiratory epithelium in COVID-19 patients, including AT2 cells, Krt5+ basal cells, and lung progenitor Tm4sf1+ and Krt5+ cells [138,139,140]. This suggests that different populations of proliferating progenitor cells are concentrated in areas of lung damage to help regenerate the trachea and alveoli after SARS-CoV-2 infection. This regeneration may also be influenced by intestinal health via the GLA, which is indirectly supported by the intestinal symptoms in COVID-19 patients (20–60%) and by the high expression of SARS-CoV-2 receptors (e.g., ACE2) in intestinal epithelial cells, as mentioned earlier [141]. These SARS-CoV-2-infected enterocytes could lead to severe intestinal dysbiosis and leaky gut, further impacting lung infection, damage, and regeneration. For instance, the potential link between gut-derived Trimethylamine N-oxide (TMAO) and lung regeneration may involve regulating vascular inflammation and endothelial damage [142]. Furthermore, the improvement observed in COVID-19 patients treated with probiotics highlights the crucial role of the gut microbiome in lung recovery, including cell regeneration and differentiation following damage, although the underlying mechanisms warrant further investigation [141,143].

### 5.2. Chronic Obstructive Pulmonary Disease (COPD)

Evidence linking the GLA to COPD pathogenesis is well established at the level of dysbiosis and systemic inflammation, but direct mechanistic evidence connecting gut-derived signals to AT2/progenitor-specific regeneration remains limited; the discussion below should be read accordingly, distinguishing established immunometabolic associations from AT2-specific extrapolations. Chronic obstructive pulmonary disease (COPD) affects over 400 to 600 million people and causes more than 3 million deaths each year, making it the third leading cause of death worldwide. It can be classified by five main risk factors: genetic predisposition, early-life events, infections, exposure to tobacco smoke, and air pollution [167,168]. Dyspnoea, chronic cough (often associated with phlegm), exercise intolerance, and episodic flare-ups of respiratory symptoms (also known as exacerbations) are common symptoms of COPD. This is due to its heterogeneous respiratory condition, characterized by injury and remodeling of the airways, lung parenchyma, and lung vasculature [168]. Although the resulting molecular endotypes may affect disease development, it is clear that the loss of lung structural integrity and regenerative capacity is a key driver of disease progression [167]. These capacities could also be influenced by the local microenvironment and by factors beyond it, including microbial composition and diversity. For instance, pulmonary pathogenic *Proteobacteria*, particularly *Haemophilus*, were more prevalent in COPD patients. In contrast, bacterial metabolites, such as adenosine, 5′-methylthioadenosine, sialic acid, tyrosine, and glutathione, are associated with a better prognosis in COPD [9,10,169]. Meanwhile, any association between COPD and the gut microbiome should be attributed to specific microbial taxa rather than to the microbiome as a whole. Increased abundances of genera such as *Faecalicatena*, *Oscillibacter*, *Lawsonibacter*, *Flavonifractor*, and *Streptomyces*, and reduced abundances of *Lachnospira*, *Eubacterium*, and *Coprococcus*, were associated with the incidence of COPD [170].

Lung regeneration is the ability of the lung to repair itself after damage through the proliferation and differentiation of resident progenitor cells, along with many structural cell types, including mesenchymal, endothelial, and epithelial cells. Specifically, epithelial progenitor cells, such as bronchial epithelial cells and AT2s, regenerate different epithelial types throughout the respiratory tract and in the alveolar space when the lung is damaged by diseases such as COPD [167]. For example, a subpopulation of AT2 cells with transcriptional evidence of abnormal cellular metabolism and decreased stress tolerance was found to be crucial to COPD development [144]. Whether the gut microbiome directly influences the regeneration of lung damage caused by COPD remains largely unknown. However, skeletal muscle wasting (or sarcopenia), common among many COPD patients, has been reported to worsen due to gut dysbiosis. This condition could lead to a loss of muscle-derived myokines and create a systemic environment that promotes cellular aging in the lungs, along with gut-derived inflammatory cytokines (e.g., TNF-α and IL-17) [39,171,172]. Also, it has been proven that cigarette-smoking-induced COPD in mice can be affected through the GLA by modifying the composition of the intestinal microbiota using antibiotics or microbiome transplantation. Furthermore, treatment with vancomycin and ampicillin or a combination, unlike other antibiotics, was associated with reductions in IL-1β and TNF-α production by F4/80+CD11b+ macrophages and IL-17A in CD4+NKp46+ Th17 cells, along with an increase in IL-10 production by Th17 cells [145]. These cytokines, such as IL-1 and TNF-α, are widely reported to contribute to the alveolar regeneration [137,146]. Therefore, it is not surprising that the gut microbiome influences lung progenitor cell regeneration during lung recovery from COPD via the GLA, although a direct gut-derived signal-to-AT2 mechanism has not been demonstrated. For instance, it was reported that cigarette smoke depletes intestinal *Bacteroidetes*, which is an important source of acetate in the gut. Acetate is one of the SCFAs and has been highlighted for its critical role in the metabolic health of lung capillary endothelium [147,148].

Beyond these established immunometabolic connections, recent single-cell and mechanistic studies suggest that AT2 cell senescence, rather than simple stem cell exhaustion, is a proximal driver of failed alveolar repair in emphysema. AT2 cells isolated from aged human lungs show measurable loss of self-renewal and differentiation capacity alongside an inflammaging transcriptional signature [173]. Also, genetic or pharmacological elimination of p16ᴵᴺᴴᵃ-expressing senescent cells has been shown to restore alveolar epithelial regenerative capacity in cigarette-smoke- and elastase-induced emphysema models [174]. As gut-derived butyrate acts as an HDAC inhibitor capable of reprogramming AT2 gene expression toward a pro-regenerative state (Section 4.2.1), and cigarette-smoke-induced depletion of intestinal *Bacteroidetes* reduces circulating acetate availability, a plausible but as yet untested hypothesis is that loss of gut-derived SCFA signaling removes an epigenetic brake on AT2 senescence in COPD, compounding its established effects on lung capillary endothelial metabolism. This hypothesis is consistent with, though not directly tested by, existing evidence that dietary fiber supplementation, which increases gut SCFA, bile acid, and sphingolipid output, attenuates emphysema progression in cigarette-smoke-exposed mice [175], and that gut-derived butyrate reduces cigarette-smoke-induced lung damage via inhibition of the mevalonate pathway in the gut, lung, and liver [26].

### 5.3. Asthma

As with COPD, most evidence linking the GLA to asthma pathogenesis is correlational or immune-focused rather than directly demonstrating effects on airway epithelial progenitor regeneration. This distinction is noted explicitly below. Asthma is a chronic respiratory disease associated with substantial illness and death, and has been diagnosed in more than 360 million people [176,177]. This disorder is prevalent worldwide, with much higher rates in high-income countries, despite its susceptibility in most individuals, and encompasses a wide spectrum of diseases characterized by shortness of breath, chest pain, wheezing, and coughing that vary in intensity over time [178,179,180]. These symptoms are primarily caused by airway inflammation, hyperresponsiveness, and airflow limitation, with a complex pathogenesis involving various genetic, environmental, and immunological factors [181,182]. It is the most common chronic inflammatory disease of the respiratory tract, characterized by leukocyte infiltration and tissue remodeling, with the latter typically referring to epithelial hyperplasia, collagen deposition, subepithelial basement membrane thickening, and fibrosis [182,183,184]. Dysbiosis of the microbiome in both the lung and the gut has been shown to underlie the pathogenesis of asthma, although most evidence is correlational rather than mechanistic. For example, an increase in pathogenic communities (e.g., *Haemophilus*, *Staphylococcus*, and *Actinomyces*) in the lung microbiota, along with a decrease in commensal bacteria (e.g., *Prevotella* and *Veillonella*) [2,185,186], and a reduced abundance of the intestinal *Akkermansia*, *Bifidobacterium*, and *Faecalibacterium* in early life has been associated with human asthma [91,149].

One of the central features of asthma is airway remodeling, including excessive proliferation of smooth muscle cells and subepithelial fibrosis, which is associated with enhanced differentiation of bronchial fibroblasts into myofibroblasts, primarily induced by transforming growth factor-β (TGF-β). These remodeling processes and later lung regeneration and recovery from asthma damage have been proposed to be influenced by GLA via the microbiome or microbial metabolites. High concentrations of *Haemophilus*, *Fusobacterium*, *Neisseriaceae*, *Sphingomonas*, and *Porphyromonas* in the trachea contribute to severe atopic asthma, whereas colonization of the intestinal tract by *Lactobacilli* and *Bifidobacteria* inversely contributes to the risk of allergy [187,188]. Moreover, 2 weeks of oral administration of *Clostridium leptum* increased the percentage and total number of Tregs in the spleen and mediastinal lymph nodes. They enhanced IL-10 and transforming growth factor-β1 (TGF-β1) production in the lungs, thereby negatively regulating asthma [150]. Interestingly, the Chinese herbal formula Tingli Dazao Xiefei Decoction (TD) was reported to ameliorate NO-CO metabolism in the lung and, indirectly, in the intestine, ultimately achieving co-regulation of lung and intestinal inflammation, immune imbalance, cellular barrier damage, oxidative stress, and intestinal bacterial disorders in asthma in vivo and in vitro [189]. Although all these studies clearly show that the lung and gut microbiomes contribute to cell proliferation, differentiation, regeneration, and recovery in asthma, the rationale behind this remains largely unknown and warrants further investigation. However, given the growing evidence for the role of the lung microbiome in the development, severity, and heterogeneity of asthma, it is reasonable to consider the microbiome a specific target for manipulation in asthma prevention and/or treatment. Current treatment modalities for asthma include bronchodilators, antibiotics, steroids, and, more recently, biologic therapy [190].

### 5.4. Idiopathic Pulmonary Fibrosis (IPF)

Among the five diseases discussed in this review, IPF has among the strongest direct evidence linking AT2 dysfunction to disease pathogenesis, although evidence specifically implicating gut-derived microbial signals in this AT2 dysfunction remains more limited and is highlighted as such below. Idiopathic pulmonary fibrosis (IPF) affects over 3 million people and increases year by year, with the most prominent non-modifiable risk factors being male gender and age [191]. It is a chronic and progressive fibrotic lung disease characterized by scarring of the interstitium of the lungs, with high mortality and limited treatment options [191,192,193]. The complex pathophysiology of IPF, underpinned by alterations across many aspects of molecular and cellular physiology, including genetics, epigenetics, microRNAs, developmental reprogramming, cell-signaling pathways, apoptosis, metabolism, autophagy, and, more recently, the microbiome of the lung and gut, makes investigation more challenging and demands a systems-level approach to understanding its pathophysiology [191]. It is currently believed that pulmonary fibrosis results from repetitive injury to the lung epithelium, leading to fibroblast accumulation, myofibroblast activation, and matrix deposition. These injuries, together with innate and adaptive immune responses, dysregulated wound repair, and fibroblast dysfunction, drive recurrent tissue remodeling and self-perpetuating fibrosis. IPF is characterized by remodeling of the interstitium, distal airway, and alveolar spaces [192]. More recently, it has been established that the gut and lung microbiomes are involved in the etiology and progression of IPF via the GLA. For example, several studies have found significant alterations in microbial composition in the lungs or guts of IPF patients compared with healthy controls [194,195,196,197,198,199].

As mentioned earlier, in IPF, repetitive micro-injuries to alveolar epithelial cells trigger abnormal epithelial–fibroblast communication, which ultimately leads to abnormal extracellular matrix (ECM) accumulation and pathological lung remodeling [193]. More recent research has highlighted the important roles of stem cell (e.g., AT2) dysfunction and the extracellular matrix in mediating lung pathological remodeling and promoting fibrosis. These AT2 dysfunctions lead to failed regeneration, profibrotic epithelial–mesenchymal crosstalk, fibroblast activation, and ECM stiffening. For instance, knockout of the ubiquitin ligase *Nedd4-2* in AT2 cells led to chronic lung disease that shared key features with IPF [151]. Conversely, intratracheal transplantation of AT2 cells or iPSC (induced pluripotent stem cell)-derived AT2 cells could reverse lung fibrosis [152,153]. Moreover, bidirectional communication between the gut and the lungs, or the GLA, mediated by interactions among microorganisms, immune functions, and metabolic products, has been proposed to play an important role in IPF. For example, changes in the gut microbiota could significantly alter the proportions of CD4+IL-6+ and CD4+IL-17A+ T cells in the lungs, thereby affecting pulmonary fibrosis by activating the IL-6/STAT3/IL-17A pathway [154]. In addition, intestinal microbial metabolites, such as amino acids, SCFAs, bile acids, and valproic acid, have also been found to be involved in IPF [200,201,202,203]. Natural-product-based, microbiota-modulating strategies are also being explored for pulmonary fibrosis: network pharmacology analyses of Astragalus polysaccharides identify multi-target, multi-pathway anti-fibrotic mechanisms relevant to IPF [204]. Separately, recent work has identified a PARP1-FOXN3-p38 feedback axis that suppresses pulmonary fibrosis by restraining profibrotic Smad signaling, illustrating a signaling mechanism regulating fibrotic remodeling whose connection to gut–lung axis signaling specifically has not yet been established [205]. Recent work has further clarified the specific AT2 mechanistic switch that determines regenerative success versus fibrotic failure in IPF. IL-11 signaling in AT2 cells has been shown to stall the beneficial AT2-to-AT1 differentiation by promoting a dysfunctional KRT8-high state and blocking IL-11 signaling, both of which prevent this stalled state and restore productive alveolar regeneration in vivo [206]. Notably, this AT2 fate switch is itself under direct epigenetic control: the let-7 microRNA family maintains AT2 quiescence and promotes AT1 differentiation, in part by regulating histone H3K27 acetylation and methylation at AT2 fibrogenic genes. And its loss is sufficient to drive uncontrolled AT2 transitional cell accumulation and fibrosis [207]. This direct evidence that AT2 fibrogenic fate is governed by histone acetylation dynamics closely parallels this review’s central argument that gut-derived, HDAC-inhibiting SCFAs such as butyrate regulate AT2 epigenetic state (Section 4.2.1), raising the specific, testable hypothesis that gut-derived epigenetic signals converge on this same let-7-regulated chromatin circuit to influence whether AT2 cells resolve toward AT1 differentiation or persist as pro-fibrotic transitional intermediates. However, the specific mechanisms by which interactions within the GLA affect progenitor cell regeneration and differentiation, as well as damage and recovery from IPF-induced injury, remain unclear.

### 5.5. Lung Cancer

Again, evidence connecting the GLA to lung cancer is expanding rapidly but remains largely correlative with respect to progenitor cell regeneration specifically, as distinct from the pathological co-option of regenerative programs during tumorigenesis. As one of the most aggressive and prevalent diseases worldwide, lung cancer accounted for 2.2 million new cases and 1.8 million deaths in 2020, more deaths than breast, colorectal, and prostate cancers combined [208,209]. It is broadly divided into two main histologic subtypes: small cell lung cancer (SCLC, 15% of cases) and non-small cell lung cancer (NSCLC, 85% of cases). NSCLC is further divided into adenocarcinoma, squamous cell carcinoma (SCC), and large cell carcinoma (LCC) [210]. Its tumor grows relatively slowly, with a later onset of invasion and metastasis compared to SCLC, which is a very aggressive malignancy with a poor prognosis and the worst pathological type of lung cancer. However, it is the least common lung cancer [210]. The process of lung cancer is quite complicated and heterogeneous, as a consequence of several genetic and epigenetic factors, particularly those associated with the activation of pathways involved in growth and inhibition [211]. These cell cycle regulation-related mutations lead to inhibited or accelerated cell proliferation and reduce the cells’ sensitivity to inhibitory signals, such as TP53, RB, and p16 [212]. Although genetics, gender, lifestyle, and environmental exposures, including biomass fuels, occupational exposures, and pollution, are risk factors for lung cancer, cigarette smoking is the most established and widely recognized risk factor, and incidence trends largely mirror regional smoking patterns [209]. More recently, symbiotic microbiota have emerged as important biomarkers and modulators of oncogenesis, including the lung and gastrointestinal tract microbiomes in the context of lung cancer. For example, lung cancer patients exhibit upregulated pulmonary *Streptococcus* and *Staphylococcus* and downregulated *Streptomyces* levels, which could lead to DNA damage, induce genomic instability, and alter host susceptibility to carcinogenic insults, thereby contributing to lung cancer development [2,213]. Similarly, in patients with lung cancer, upregulation of *Bacillus* and *Akkermansia muciniphila*, and downregulation of *Bifidobacterium* and *Faecalibacterium* in the gut microbiota were found to influence tumorigenesis by altering TNF-a- and LPS-induced inflammation [39,214,215].

Tumors have long been suspected of hijacking stem cell mechanisms that support tissue maintenance and repair. For instance, club cells in the airway, BASCs (bronchoalveolar stem cells), and AT2s in the alveoli have all been found to play a critical role in lung cancer tumorigenesis [155,216,217]. For example, *KrasG12D*-expressing mutant AT2 cells co-opt a regeneration program during tumorigenesis by affecting equivalent proliferative potential across heterogeneous mutant states, thereby driving oncogenic expansion through the *Il1r1* and NF-kB program [155]. It is important to note that this oncogenic co-option of AT2 regenerative programs represents a pathological subversion of proliferative signaling, mechanistically distinct from the reparative AT2 regeneration discussed elsewhere in this review: the same plasticity that enables productive alveolar repair can, under oncogenic mutation, be redirected toward tumorigenic expansion rather than restorative differentiation. This oncogenic co-option of AT2 plasticity is now being resolved at higher molecular resolution. Single-cell multi-omic profiling of *Kras/p53*-driven tumor organoids shows that AT2-like tumor cells retain distinct chromatin and transcription factor programs from more dedifferentiated, EMT-associated tumor states. Meanwhile, AT2-like tumor cells with these regulatory programs intact show greater tumorigenic potential in vivo, identifying specific chromatin-level dependencies of the AT2-like tumor state as candidate early-intervention targets [218]. Whether gut-derived metabolic and epigenetic signals (Section 4.2) influence which regulatory programs oncogenically initiated AT2 cells adopt remains unknown. But it represents a direct, testable extension of the AT2-centered framework proposed throughout this review to the earliest stages of lung tumorigenesis. Moreover, these progenitor activities could also be affected by the microbiomes of both the local lung tissue and the intestinal tract. It was reported that intratumoral *Roseburia*, a well-known butyrate-producing bacterium, could promote lung cancer metastasis, while butyrate could directly promote lung cancer cell invasion by increasing H19 and MMP15 expression and inducing M2 macrophage polarization [156]. Meanwhile, gut microbiome alterations resulting from *Lcn2* loss fostered the proliferation of proinflammatory bacteria of the genus *Alistipes*, triggering gut inflammation that propagated systemically, inducing immunosuppression within the tumor microenvironment, augmenting tumor growth through an IL6-dependent mechanism, and dampening the response to immunotherapy [157]. However, how these microbiota, directly and indirectly via their products, affect lung cancer tissue damage and regeneration, especially in progenitor cells, remains largely unknown and requires further investigation. Clarifying these mechanisms can help us better understand the importance of the GLA in lung cancer pathogenesis and improve current treatments and future therapies.

## 6. Conclusions and Future Directions

The paradigm of pulmonary medicine has undergone a transformative shift, recognizing that the lungs do not function in isolation. The GLA serves as a vital physiological bridge linking distal microbial ecology with local respiratory homeostasis. As detailed in this review, the intestinal microbiota functions as a “bioreactor,” converting dietary inputs into a systemic pharmacopeia of metabolites, including SCFAs, indole derivatives, and secondary bile acids, that calibrate the threshold for inflammatory activation and the efficiency of lung regeneration. Moreover, the GLA operates as a double-edged sword. In a state of eubiosis, it provides the “basal tonicity” required for immune surveillance and the metabolic fuel necessary for progenitor cell fitness. However, in the context of critical illness, such as ARDS or severe COVID-19, the axis becomes a “pathological circuit.” The breakdown of the intestinal barrier transforms the gut into a source of toxic lymph and translocated PAMPs, which bypass the liver and directly assault the pulmonary microvasculature. This transition from a homeostatic partner to a driver of multi-organ failure underscores the gut’s role as the “motor” of critical illness. Here, a central theme of this review is that successful recovery from lung injury is not merely the absence of inflammation but the active completion of lung regeneration. We have highlighted that the AT2 cell niche is highly sensitive to gut-derived signals. Microbial metabolites could act as epigenetic and metabolic switches that determine whether an AT2 cell differentiates to restore gas exchange or enters a senescent state that leads to fibrosis. Consequently, persistent pulmonary symptoms in chronic diseases like COPD and IPF may reflect a state of “metabolic starvation” in which the lung lacks the microbial cues necessary to complete its regenerative program.

Beyond conventional probiotics, an expanding translational toolkit is emerging for gut-targeted intervention in lung disease. Next-generation probiotics and engineered bacterial therapeutics designed to deliver specific metabolic outputs (e.g., defined SCFA-producing consortia), direct microbial metabolite supplementation, bacteriophage-based microbiome modulation, and precision, biomarker-guided FMT protocols each represent strategies that move beyond broad-spectrum microbiota manipulation toward targeted restoration of the specific gut-derived signals discussed throughout this review [219]. Complementary inflammation-targeted approaches, such as engineered cellular vesicle platforms designed to suppress pulmonary inflammation and promote recovery, further illustrate the breadth of emerging strategies at the gut-lung interface [220].

Beyond therapeutic strategy, methodological advances are similarly reshaping how the AT2-centered framework proposed in this review can be directly tested. Spatial transcriptomic approaches can now resolve epithelial cell states and their local microenvironment at single-cell resolution within intact lung tissue. For example, image-based spatial transcriptomics of over 1.6 million cells across human control and fibrotic lungs has identified discrete molecular niches marking the transition from healthy to dysregulated alveolar epithelium, a resolution unattainable with bulk sequencing approaches [221]. In parallel, spatial host-microbiome sequencing techniques now allow simultaneous capture of host transcriptomes and bacterial taxa within the same tissue section, directly visualizing host-microbiome spatial niches in mucosal barrier tissues such as the gut [222]. Applying such integrated single-cell, spatial transcriptomic, and spatial microbiome profiling approaches directly to the lung and gut in tandem represents an important methodological frontier for testing whether gut-derived signals discussed throughout this review causally shape AT2 cell fate in situ, rather than relying solely on correlative or bulk-tissue evidence.

Therefore, the GLA is a frontier in precision medicine. By shifting our focus from the lung as an autonomous organ to the lung as part of a systemic microbial–host network, we open new avenues for therapeutic interventions. Future strategies that target the “gut end” of the axis, such as precision probiotics, postbiotic supplementation, or fecal microbiota transplantation (FMT), could revolutionize how we treat refractory lung damage and promote robust lung regeneration in the face of increasingly complex global respiratory threats [223].

Looking ahead, the GLA is no longer best understood as a one-directional inflammatory link between the intestine and the airways. Instead, it should be viewed as a bidirectional regulatory network that integrates immune trafficking, microbial metabolites, epithelial barrier integrity, and direct microbial dissemination to shape lung homeostasis and repair. In this review, we have emphasized that the AT2 cell niche is particularly sensitive to gut-derived signals and may serve as a key determinant of whether injured lung tissue regenerates successfully or progresses toward persistent dysfunction and fibrosis. This framework also highlights several important gaps. Much of the current evidence remains associative, and future studies should prioritize causal designs, longitudinal human cohorts, spatial multi-omics, and AT2-focused experimental systems. Mechanistic work is needed to define which microbial signals are protective, which are harmful, and which act in a context-dependent manner across COVID-19, COPD, asthma, IPF, and lung cancer. Ultimately, precision microbiome-based strategies, including targeted probiotics, postbiotic supplementation, dietary modulation, and FMT, may serve as useful adjuncts to promote lung repair and restore epithelial resilience.

## Figures and Tables

**Table 1:** Gut-derived signals in the gut–lung axis.

Signal/Metabolite Class	Microbial Source	Representative Molecules	Receptor/Target	Downstream Pathway	Reported Effect on AT2/Progenitor Regeneration	Disease Context	Therapeutic Implications	References
SCFAs (physiologic)	Fiber-fermenting *Firmicutes* (e.g., *Faecalibacterium*, *Roseburia*) and *Bacteroidetes*	Acetate, propionate, butyrate	GPR41/FFAR3, GPR43/FFAR2, GPR109A/HCAR2	GPCR (Gi-protein) signaling, leading to cAMP/PKA modulation; butyrate additionally acts as an HDAC inhibitor (histone hyperacetylation)	Anti-inflammatory GPCR signaling; butyrate (HDAC inhibitor) linked to pro-regenerative AT2 gene expression; acetate restores fatty-acid oxidation/acetyl-CoA needed for AT2 regeneration	Acute lung injury, asthma, COPD	Dietary fiber/prebiotic supplementation; SCFA-producing probiotic consortia; direct butyrate/propionate supplementation	[24,53,54,55]
SCFAs (supraphysiologic)	Same fiber-fermenting taxa as above, at excessive local concentration	Butyrate, propionate (10–25 mM)	FFAR3/p38 MAPK	FFAR3-p38 MAPK, resulting in NF-κB-mediated IL-6/CXCL8 induction	Paradoxically pro-inflammatory: potentiates TNF-α-induced IL-6/CXCL8 in lung fibroblasts & airway smooth muscle	In vitro human lung fibroblasts/ASM	Highlights need for concentration-controlled, targeted delivery rather than bulk SCFA supplementation	[56]
Propionate (systemic)	Propionate-producing *Bacteroidetes* and *Negativicutes*	Propionate	Not receptor-specific (bone marrow DC precursor effect)	Reprogramming of DC hematopoiesis (receptor-independent mechanism reported)	Skews dendritic-cell hematopoiesis toward a non-allergic phenotype	Allergic airway models	Maternal/early-life propionate supplementation to modulate offspring allergic-airway risk	[57]
Secondary bile acids (systemic)	*Clostridium scindens* and related 7α-dehydroxylating *Firmicutes*	Deoxycholic acid (DCA), lithocholic acid (LCA)	FXR, TGR5	FXR/TGR5, leading to NF-κB inhibition and anti-fibrotic gene programs	Anti-inflammatory, anti-fibrotic; reduced vascular remodeling	Pulmonary hypertension, pulmonary fibrosis (experimental)	FXR/TGR5 agonists as candidate anti-fibrotic/anti-remodeling agents in pulmonary hypertension and IPF	[58,59]
Bile acids (local/microaspiration)	Same colonic bile-acid-modifying bacteria; reaches lung via reflux/aspiration rather than circulation	LCA, DCA, chenodeoxycholic acid (CDCA)	FXR-dependent & independent pathways	FXR-dependent and -independent epithelial injury/EMT signaling	Alveolar epithelial injury, epithelial–mesenchymal transition, fibroblast activation	IPF (in vitro/ex vivo)	Reflux/aspiration prevention (e.g., post-lung-transplant management) rather than direct bile acid modulation	[60]
Tryptophan/indole metabolites	*Lactobacillus* spp. and other tryptophan-metabolizing commensals	Indole-3-aldehyde and related AhR ligands	Aryl hydrocarbon receptor (AhR)	AhR, leading to HDAC5/6–NF-κB–NLRP3 inhibition; IL-22 induction	Barrier integrity, IL-22 production, antimicrobial defense; HDAC5/6-NF-κB-NLRP3 inhibition reduces inflammation; preserves alveolar epithelial identity and limits TGF-β-driven epithelial-to-mesenchymal transition during injury	COPD, cystic fibrosis, Pseudomonas lung infection, ARDS	AhR-ligand-producing probiotics; dietary tryptophan/indole supplementation	[61,62,63,64,65,66,67,68,69]
Bacterial extracellular vesicles (BEVs)	*Bifidobacterium* and other commensal/probiotic species	Commensal/probiotic BEVs (e.g., *Bifidobacterium*)	Not fully characterized	Implicated in CD8+ T-cell priming and PD-1 pathway modulation	Increased CD8 T-cell infiltration, enhanced anti-PD-1 efficacy; tolerogenic lung immune landscape	Lung tumors, infection resistance	BEV-based immunotherapy adjuncts; engineered BEV delivery platforms	[70,71,72]
LPS/PAMPs (via BEVs or direct translocation)	Gram-negative *Enterobacteriaceae* and other gut pathobionts	Lipopolysaccharide (Gram-negative bacterial wall)	TLR4/MyD88/NF-κB	TLR4/MyD88, resulting in NF-κB-driven pro-inflammatory transcription	Pro-inflammatory; drives ARDS/severe COVID-19 pathology; OSM induction in severe asthma	ARDS, severe COVID-19, severe asthma	TLR4 antagonists; gut barrier reinforcement to limit LPS translocation	[27,73,74,75,76,77,78,79]

**Table 2:** Representative studies relevant to the proposed GLA-AT2 regeneration framework.

Disease	Model/System	Key Finding Relevant to Progenitor/AT2 Regeneration	References
COVID-19/ARDS	Human patients; ex vivo lung tissue	Lung microbial burden and TNF-α/IL-1β linked to AT2 proliferation/differentiation; AT2, Krt5+ basal, and Tm4sf1+ progenitors mobilize in damaged epithelium	[133,134,135,136,137,138,139,140]
COVID-19	Human patients (GI-symptom cohort)	Gut-derived TMAO implicated in vascular inflammation/endothelial damage affecting regeneration; probiotic treatment improves recovery	[141,142,143]
COPD	Human COPD cohort	AT2 subpopulation with abnormal metabolism/reduced stress tolerance identified as crucial to COPD; direct gut-microbiome-to-AT2 link remains unestablished	[144]
	Mouse (cigarette smoke + antibiotics/microbiome transplant)	Antibiotic-driven gut microbiota shifts alter macrophage/Th17 cytokine profiles implicated in alveolar regeneration	[145,146]
	Human/animal (cigarette smoke exposure)	Cigarette smoke depletes gut *Bacteroidetes* (an acetate source); acetate implicated in lung capillary endothelial metabolic health	[147,148]
Asthma	Human early-life cohorts	Reduced *Akkermansia*, *Bifidobacterium*, *Faecalibacterium* in early life associated with asthma risk (largely correlative)	[91,149]
	Mouse (oral *Clostridium leptum*)	Increased Treg numbers and IL-10/TGF-β1 in the lung; negatively regulate asthma	[150]
IPF	Mouse (*Nedd4-2* knockout in AT2 cells)	AT2-specific gene knockout produces IPF-like chronic lung disease, establishing AT2 dysfunction as causal	[151]
	Mouse/iPSC-derived AT2 models	AT2 or iPSC-derived AT2 cell transplantation reverses lung fibrosis	[152,153]
	Human/mouse	Gut microbiota alterations shift CD4+IL-6+/IL-17A+ T-cell proportions, activating the IL-6/STAT3/IL-17A fibrotic pathway	[154]
Lung cancer	Mouse (*KrasG12D*)	Mutant AT2 cells co-opt a regeneration program for tumorigenesis via Il1r1/NF-κB (pathological, mechanistically distinct from reparative regeneration)	[155]
	Mouse (intratumoral *Roseburia*)	Butyrate-producing bacteria promote metastasis via H19/MMP15 upregulation and M2 macrophage polarization	[156]
	Mouse (*Lcn2* knockout)	Gut *Alistipes* expansion drives systemic inflammation and immunosuppression, augmenting tumor growth via an IL-6-dependent mechanism	[157]

## Data Availability

No new data were created or analyzed in this study. Data sharing is not applicable to this article.
